# Organokines and liver enzymes in adolescent girls with polycystic ovary syndrome during randomized treatments

**DOI:** 10.3389/fendo.2024.1325230

**Published:** 2024-05-16

**Authors:** Cristina Garcia-Beltran, Marion Peyrou, Artur Navarro-Gascon, Abel López-Bermejo, Francis de Zegher, Francesc Villarroya, Lourdes Ibáñez

**Affiliations:** ^1^ Endocrinology Department, Institut de Recerca Sant Joan de Déu, University of Barcelona, Barcelona, Spain; ^2^ Centro de Investigación Biomédica en Red de Diabetes y Enfermedades Metabólicas Asociadas (CIBERDEM), Instituto de Salud Carlos III, Madrid, Spain; ^3^ Biochemistry and Molecular Biomedicine Department, Biomedicine Institute, University of Barcelona, Barcelona, Spain; ^4^ Centro de Investigación Biomédica en Red de Fisiopatología de la Obesidad y Nutrición (CIBEROBN), Instituto de Salud Carlos III, Madrid, Spain; ^5^ Pediatric Endocrinology Research Group, Girona Institute for Biomedical Research (IDIBGI), Faculty of Medicine, University of Girona and Dr. Josep Trueta Hospital, Girona, Spain; ^6^ Leuven Research and Development, University of Leuven, Leuven, Belgium

**Keywords:** organokines, PCOS, spironolactone, pioglitazone, metformin, oral contraceptives, METRNL, liver enzymes

## Abstract

**Introduction:**

Polycystic ovary syndrome (PCOS) is often associated with metabolic-associated fatty liver disease (MAFLD). MAFLD has been associated with altered hepatic function, systemic dysmetabolism, and abnormal circulating levels of signaling molecules called organokines. Here, we assessed the effects of two randomized treatments on a set of organokines in adolescent girls with PCOS and without obesity, and report the associations with circulating biomarkers of liver damage, which were assessed longitudinally in the aforementioned studies as safety markers.

**Materials and methods:**

Liver enzymes [aspartate aminotransferase (AST), alanine aminotransferase (ALT), and gamma-glutamyl transferase (GGT)] were assessed as safety markers in previous randomized pilot studies comparing the effects of an oral contraceptive (OC) with those of a low-dose combination of spironolactone-pioglitazone-metformin (spiomet) for 1 year. As a *post hoc* endpoint, the organokines fibroblast growth factor-21 (FGF21), diazepam-binding protein-1 (DBI), and meteorin-like protein (METRNL) were assessed by ELISA after 6 months of OC (N = 26) or spiomet (N = 28). Auxological, endocrine-metabolic, body composition (using DXA), and abdominal fat partitioning (using MRI) were also evaluated. Healthy, age-matched adolescent girls (N = 17) served as controls.

**Results:**

Circulating ALT and GGT levels increased during OC treatment and returned to baseline concentrations in the post-treatment phase; in contrast, spiomet treatment elicited no detectable changes in ALT and GGT concentrations. In relation to organokines after 6 months of treatment, (1) FGF21 levels were significantly higher in PCOS adolescents than in control girls; (2) DBI levels were lower in OC-treated girls than in controls and spiomet-treated girls; and (3) no differences were observed in METRNL concentrations between PCOS girls and controls. Serum ALT and GGT levels were directly correlated with circulating METRNL levels only in OC-treated girls (R = 0.449, P = 0.036 and R = 0.552, P = 0.004, respectively).

**Conclusion:**

The on-treatment increase in ALT and GGT levels occurring only in OC-treated girls is associated with circulating METRNL levels, suggesting enhanced METRNL synthesis as a reaction to the hepatic changes elicited by OC treatment.

**Clinical Trial Registration:**

https://doi.org, identifiers 10.1186/ISRCTN29234515, 10.1186/ISRCTN11062950.

## 1 Introduction

Polycystic Ovary Syndrome (PCOS) is a common condition in adolescents and young women (1, 2). Adolescent PCOS is characterized by a combination of clinical and/or biochemical androgen excess and anovulatory oligo-amenorrhea that presents between 2 and 8 years after menarche (3). The entity appears to be driven by ectopic fat accumulation, particularly in the liver, leading to insulin resistance (3), and by reduced energy expenditure, partly due to a lower activity of brown adipose tissue (4), favoring weight gain, and increasing the risk for type 2 diabetes (5).

PCOS in adolescents and women is often associated with non-alcoholic fatty liver disease (NAFLD) (6, 7). Insulin resistance and hyperandrogenemia have been found to be major contributory factors independent of body mass index (BMI) (7). Genetically predicted NAFLD is associated with a higher risk of PCOS, as judged by bidirectional two-sample Mendelian randomization analyses (8). Mitochondrial dysfunction, gut microbiome dysbiosis, and endocannabinoid system overactivation are among the proposed molecular mechanisms linking NAFDL and PCOS (9). NAFLD is currently considered a systemic disorder in which hepatic steatosis is merely the landmark of systemic metabolic dysfunction, including insulin resistance, increased cardiovascular risk, and low-grade systemic inflammation (10, 11). This notion has recently led to the expansion of the designation of NAFLD to “metabolically associated fatty liver disease” or MAFLD (11).

Studies in adults affected by MAFLD indicate a close association between altered hepatic function and systemic dysmetabolism, encompassing a pathogenic rearrangement of circulating signaling molecules, the so-called organokines (12), which originate in the liver (hepatokines) and adipose tissue (adipokines) or other organs and tissues (13). The altered circulating levels of these signaling molecules generate multiorgan systemic disturbances and provide biomarker evidence of existing health risks in patients at distinct stages of disease progression (14). Especifically in relation to MAFLD, emerging data indicate that an altered secretion of organokines plays an essential role in the pathogenesis of insulin resistance and cardiovascular diseases. For example, fetuin-A, a hepatokine that elicits low-grade inflammation in MAFLD by acting as an endogenous ligand of toll-like receptor-4 and promotes the secretion of proinflammatory cytokines in adipose tissue and other organs (15). Fetuin-A also suppresses the expression of the insulin-sensitizing adipokine adiponectin, which leads to systemic insulin resistance. In MAFLD, increased pro-inflammatory cytokines and enhanced levels of hepatokines such as angiopoietin-like proteins, also promote endothelial dysfunction, dyslipidemia, and atherogenesis (16). However, comprehensive knowledge of the entire set of organokines involved in linking MAFLD to systemic alterations and the associated mechanisms of action is still lacking.

Women with PCOS show altered levels of organokine signaling molecules (17, 18). In adolescent PCOS, abnormal organokine concentrations [i.e., high molecular weight adiponectin (19, 20), growth-and-differentiation factor-15 (21), fetuin-A (22), and chemokine ligand-14 (23)], have also been associated with earlier stages of hepatic and metabolic systemic alterations, even in the absence of overt obesity.

Currently, there is no approved pharmacological treatment for adolescent PCOS. The usually recommended off-label medication is an oral estroprogestagen contraceptive (OC) that is primarily used to revert androgen excess and restore menstrual regularity (24). However, this approach has a limited capacity to improve metabolic status (3, 25, 26) and may cause sustained unfavorable changes in hepatic markers (25, 27). Currently, the research in progress focuses on the development of safer medications that reduce ectopic fat and/or increase energy expenditure (3). In adolescents with PCOS and without obesity, a low-dose combination of one mixed antiandrogen and anti-mineralocorticoid (spironolactone) which increases brown adipose tissue activity (23, 28), and two insulin sensitizers (pioglitazone plus metformin) (spiomet) results in a better improvement in the metabolic condition as compared to OCs, including increased insulin sensitivity, reduced inflammation and liver fat accumulation, and more normalization of circulating hepatokines (20–23, 29). However, it is unclear to what extent the targets of spiomet include extrahepatic tissues, as well as the effects of randomized treatments on new bioactive organokines in young girls.

Existing knowledge on the identity and role of organokines connecting MAFLD hepatic disturbances with systemic metabolic and cardiovascular diseases is still limited, and there are a number of recently recognized circulating molecules that potentially play this role. We chose to analyze the effects of OCs vs spiomet on organokines recently related to MAFLD, such as meteorin-like protein (METRNL), recently reported to be related to liver injury (30); fibroblast growth factor-21 (FGF21), a hepatokine with enhanced expression in liver disease, potentially protective against systemic dysmetabolism in MALFD (31), and diazepam-binding protein-1 (DBI, also named acyl CoA-binding protein), whose blockage has been reported to improve MAFLD in recent experimental settings (32). We described their associations with circulating biomarkers of hepatic damage, which were assessed longitudinally in the aforementioned studies as safety markers (19, 20).

## 2 Materials and methods

### 2.1 Study population and design

The study population consisted of 54 adolescent girls with PCOS and without obesity [age, 16.3 ± 0.2 yr; BMI, 24.1 ± 0.5 Kg/m^2^], who participated in two randomized, open-label, pilot studies with the same design (Study 1, ISRCTN29234515 and Study 2, ISRCTN11062950, **Supplementary Figure 1**), comparing on-treatment (over 1 year) and post-treatment (over 1 year) effects of OC versus spiomet; the primary endpoint was ovulation rate after OC or spiomet intervention (19, 20). The trials were performed at the Endocrinology Department of Sant Joan de Déu University Hospital, Barcelona, Spain and the pooled results have been previously reported in detail, including the primary endpoint and secondary endpoints, namely, hirsutism score, androgens, carotid intima-media thickness, body composition, abdominal fat distribution, and hepatic fat. Serum aspartate aminotransferase (AST), alanine aminotransferase (ALT), and gamma-glutamyl transferase (GGT) levels were assessed as pre-, on-, and post-treatment safety markers (19, 20).

Due to the limited availability of spare serum, the present report focuses on 6-month on-treatment assessments. At this time point, serum was available for METRNL and DBI measurements in 33 out of the 34 randomized girls with complete data (97%) in Study 1 and in 21 out of 28 girls with complete data (75%) in Study 2, while FGF21 measurement could be performed in 30 (88%) and 20 (75%) patients, respectively. All studied patients finalized the treatment and post-treatment phases of the trials and had complete longitudinal data (**Supplementary Figure 1**).

The inclusion and exclusion criteria have been previously described in detail (19, 20). OC treatment consisted of 20 μg ethinylestradiol plus 100 mg levonorgestrel (21/28 days), and placebo (7/28 days); spiomet is a low-dose combination of spironolactone 50 mg, pioglitazone 7.5 mg, and metformin 850 mg, taken together, once daily at dinner time. A total of 17 age-matched, healthy girls recruited in nearby schools for the original studies (19, 20) in whom a spare sample was available served as controls; all had regular menses and a gynecological age >2.0 years and none were hirsute or taking medications.

### 2.2 Clinical and endocrine-metabolic assessments

Height, weight, and BMI were retrieved from medical records. Blood sampling for the assessment of endocrine-metabolic and safety parameters was performed in the early morning, after an overnight fast, in the follicular phase (days 3–7) of the cycle or after two months of amenorrhea.

Circulating testosterone was measured by liquid chromatography-tandem mass spectrometry, as described previously (19, 20); sex hormone-binding globulin (SHBG) was assessed by immunochemiluminiscence (Immulite 2000, Diagnostic Products, Los Angeles, USA), and the intra- and inter-assay coefficients of variation (CVs) were <0.5% and <8%, respectively. The free androgen index (FAI) was calculated as the ratio of serum testosterone (nmol/L) to that of SHBG (nmol/L) ×100. Serum glucose was measured by the glucose oxidase method; circulating insulin was assessed by immunochemiluminiscence (Immulite 2000, Diagnostic Products, Los Angeles, USA); intra- and inter-assay CVs were <0.1% and <7.2%, respectively. Homeostasis model assessment of insulin resistance (HOMA-IR) was calculated as [fasting insulin (mU/L)] × [fasting glucose (mg/dL)]/405. Serum AST, ALT, and GGT were assessed by molecular absorption spectrometry. Ultrasensitive C-reactive protein (us-CRP) was measured using a highly sensitive method (Architect c8000; Abbott, Wiesbaden, Germany); the intra- and inter-assay CVs were <1% and <5%, respectively.

Serum METRNL levels were assessed with a specific human enzyme-linked immunosorbent assay (ELISA) (R&D Systems, Minneapolis, MN, USA), sensitivity: 0.64 ng/mL; intra- and inter-assay CVs were <10% and <12%, respectively] (33). Serum concentrations of DBI were assessed using ELISA (Abnova, Taipei, Taiwan); intra- and inter-assay CVs were <9% (34). Circulating FGF21 levels were determined using a specific non-cross-reactive ELISA kit (Biovendor, Brno, Czech Republic), and intra- and inter-assay CVs were 3.5% and 3.7%, respectively (35).

Body composition was assessed using dual X-ray absorptiometry (DXA) with the Lunar Prodigy and Lunar software (version 3.4/3.5, Lunar Corp, WI) (15, 16). Abdominal fat partitioning (subcutaneous and visceral) and hepatic fat were assessed by magnetic resonance imaging (MRI) using a multiple‐slice MRI 1.5 T scan (Signa LX Echo Speed Plus Excite, General Electric, Milwaukee, Wisconsin, USA), as previously described (19, 20).

### 2.3 Statistics and ethics

Statistical analyses were performed using SPSS version 27.0 (SPSS software, IBM Corp., Armonk, NY, USA) and GraphPad Prism 5 (GraphPad Software, CA, USA). Results are shown as the mean ± standard error of the mean. Variables with a normal distribution were compared using two-tailed Student’s t-test. When necessary, logarithmic transformation was used to achieve a normal distribution of continuous variables. Correlations and stepwise multi-regression analysis were used to study associations between liver enzymes (AST, ALT, and GGT) and study variables, and between organokines (METRNL, FGF21, and DBI) and study variables. A covariance analysis was used to adjust for BMI. Statistical significance was set at p-value <0.05.

The study was approved by the Institutional Review Board of the Sant Joan de Déu University Hospital. Written informed consent was obtained from the parents and assent of each of the participating girls.

## 3 Results

### 3.1 Key variables in PCOS-treated girls vs. controls

The auxological, endocrine-metabolic, and imaging results in both patients and controls are shown in **Table 1**. As previously described (19, 20), spiomet intervention was associated with more normalizing effects than OC, as judged by fasting insulin, HOMA-IR, us-CRP, and hepato-visceral fat.

**Table 1:** Study variables in healthy control girls and girls with polycystic ovary syndrome (PCOS) without obesity who were randomized to receive an oral contraceptive (OC) or a low-dose combination of spironolactone plus pioglitazone plus metformin (spiomet) for 6 months.

	Controls	PCOS (6 months on-treatment)	
	(N = 17)	OC (N = 26)	spiomet (N = 28)
Auxology			
Age (years)	16.1 ± 0.3	16.3 ± 0.3	16.1 ± 0.3
Weight (kg)	58.1 ± 1.5	62.9 ± 2.3	60.1 ± 1.8
BMI (kg/m^2^)	21.8 ± 0.6	24.5 ± 0.7	23.6 ± 0.6
Endocrine—Metabolic Variables			
Testosterone (nmol/L)	1.0 ± 0.1	1.0 ± 0.1	1.1 ± 0.1
SHBG (nmol/L)	64 ± 7	63 ± 6	32 ± 2 ^c f^
FAI	1.9 ± 0.3	1.8 ± 0.2	4.2 ± 0.4 ^b f^
Glucose (mmol/L)	4.6 ± 0.3	4.8 ± 0.1	4.7 ± 0.1
Fasting insulin (pmol/L)	67 ± 8	100 ± 8	55 ± 6 ^f^
HOMA-IR	1.9 ± 0.2	3.2 ± 0.3	1.7 ± 0.2 ^f^
AST (µkat/L)	0.32 ± 0.01	0.26 ± 0.01 ^b^	0.28 ± 0.01
ALT (µkat/L)	0.26 ± 0.01	0.28 ± 0.02	0.24 ± 0.02
GGT (µkat/L)	0.21 ± 0.01	0.25 ± 0.01	0.18 ± 0.01 a ^f^
usCRP (nmol/L)	6.4 ± 1.2	22.7 ± 3.3 ^b^	5.7 ± 1.1 ^f^
METRNL (pg/mL)	369.7 ± 31.5	384.8 ± 26.7	437.4 ± 32.4
log DBI (ng/mL)	2.43 ± 0.04	2.17 ± 0.04 ^c^	2.34 ± 0.05 ^d^
log FGF21 (pg/mL)^&^	1.27 ± 0.07	1.53 ± 0.09 ^b^	1.67 ± 0.06 ^c^
Body composition (DXA) ^‡^			
Lean mass (kg)	–	37 ± 1	36 ± 1
Fat mass (kg)	–	23 ± 2	22 ± 2
Abdominal fat (kg)	–	6.2 ± 0.4	5.5 ± 0.4
Abdominal fat partitioning (MRI)			
Subcutaneous fat (cm^2^)	111 ± 13	175 ± 19	159 ± 19
Visceral fat (cm^2^)	29 ± 2	45 ± 4 ^a^	35 ± 2 ^d^
Hepatic fat (%)	10 ± 1	20 ± 1 ^c^	12 ± 1 ^f^

Values are mean ± standard error of the mean (SEM).

ALT, alanine transaminase; AST, aspartate transaminase; BMI, body mass index; DBI, diazepam-binding inhibitor; DXA, dual X-ray absorptiometry; FAI, free androgen index; FGF21, fibroblast growth factor 21; GGT, gamma-glutamyl transferase; HOMA-IR, homeostasis model assessment insulin resistance; METRNL, meteorin-like; MRI, magnetic resonance imaging; OC, oral contraceptive; SHBG, sex hormone-binding globulin; spiomet, spironolactone plus pioglitazone plus metformin; usCRP, ultrasensitive C-reactive protein.

^‡^Indicative DXA values in healthy adolescents, matched for age and height (N = 41): lean mass 35.1 ± 1.0 kg; fat mass 17.6 ± 1.4 kg (doi.org/10.1210/jendso/bvaa032).

^&^FGF21 assessment was performed in N = 24 girls on OCs and in N = 26 girls on spiomet ^a^P ^<^0.05; ^b^P ^<^0.01 and ^c^P ^<^0.001 vs controls.

^d^P ^<^0.05; ^e^P ^<^0.01 and ^f^P ^<^0.001 for differences between randomized subgroups.

P values are adjusted for BMI.

The table depicts the study variables after 6 months of active treatment.

### 3.2 Longitudinal results of liver enzymes in PCOS-treated girls

**Figure 1:**
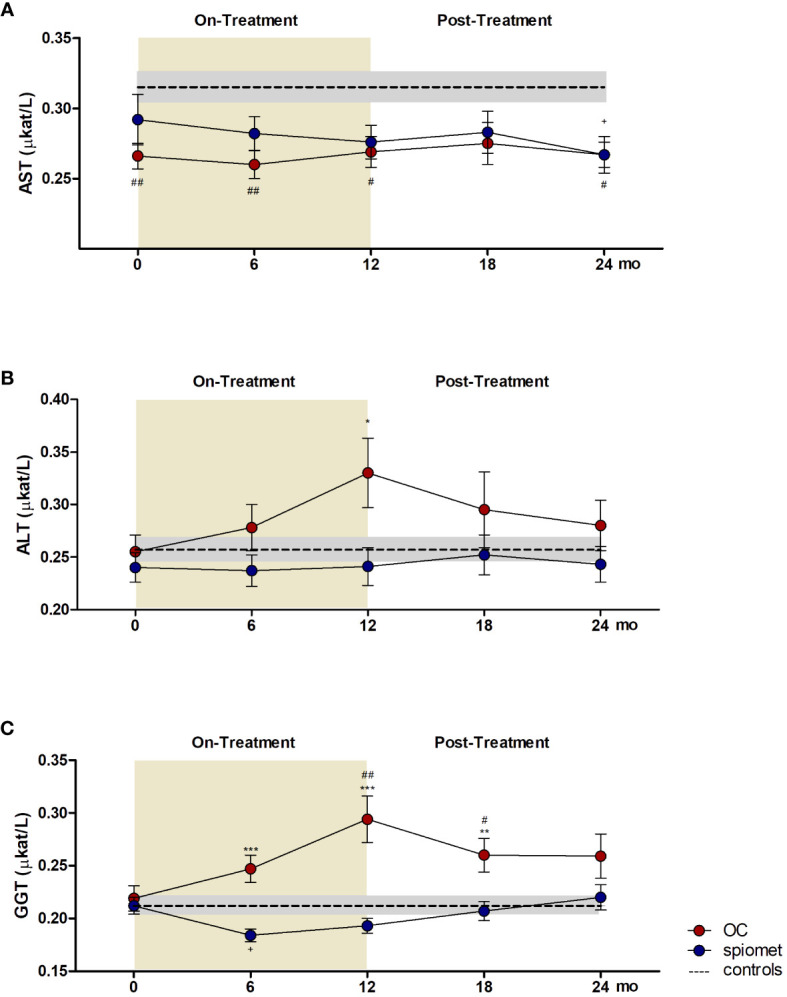
Longitudinal results of aspartate aminotransferase (AST, **A**), alanine aminotransferase (ALT, **B**), and gamma-glutamyl transferase (GGT, **C**) concentrations in adolescent girls with polycystic ovary syndrome (PCOS) who received an oral contraceptive (OC, red circles, N = 26) or a low-dose combination of spironolactone–pioglitazone–metformin (spiomet, blue circles, N = 28) for 12 months and remained untreated for 12 months. The yellow area represents the active treatment phase. The dotted line represents the mean value in healthy controls (N = 17), and the shaded area represents the mean ± standard error in healthy controls. ^*^P <0.05; ^**^P <0.01; ^***^P <0.001 for differences between subgroups at 6, 12, and 18 months. ^#^P <0.05; ^##^P <0.01, differences between controls and the OC subgroup. ^+^P <0.05, for differences between controls and spiomet subgroup.

The longitudinal results of liver enzymes (AST, ALT, and GGT) are shown in **Figure 1**. The AST levels on- and post-treatment (**Figure 1A**) were lower than those in the controls in both study subgroups. In contrast, on-treatment ALT (**Figure 1B**) and GGT (**Figure 1C**) levels were significantly increased in patients receiving OCs and remained unchanged on spiomet. After treatment, ALT and GGT concentrations decreased in the OC-treated girls, reaching levels similar to those in the control and spiomet-treated girls.

### 3.3 Organokine levels in PCOS-treated girls vs. controls

Regarding organokines (**Table 1**), on-treatment FGF21 levels were significantly increased in both PCOS subgroups compared with those in control girls. Circulating DBI levels were lower in the OC-treated girls than in the spiomet-treated girls and controls. Lastly, no differences were observed in METRNL levels between the controls and OC- or spiomet-treated girls.

### 3.4 Associations among liver enzymes, organokines, and study variables

**Figure 2:**
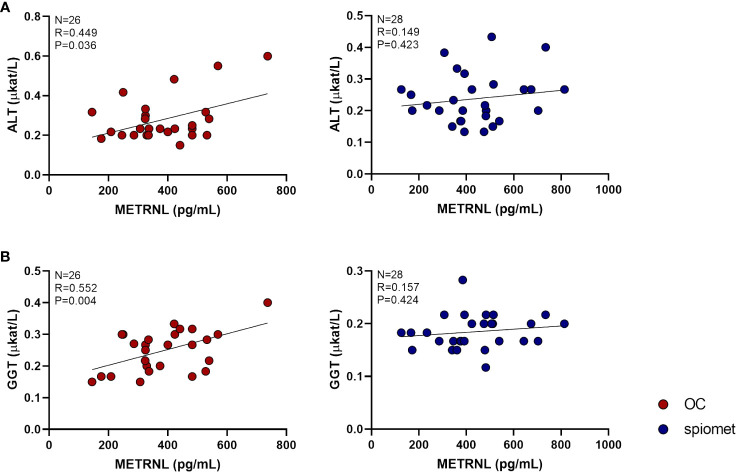
Correlations between circulating meteorin-like (METRNL) levels and alanine aminotransferase (ALT, **A**) and gamma-glutamyl transferase (GGT, **B**) concentrations in adolescent girls with polycystic ovary syndrome (PCOS) after 6 months of treatment with an oral contraceptive (OC, red circles, N = 26) or a low-dose combination of spironolactone–pioglitazone–metformin (spiomet, blue circles, N = 28). P-values were adjusted for body mass index.

The associations between liver enzymes and endocrine–metabolic, body composition, and abdominal fat partitioning variables after 6 months of OC or spiomet treatment are shown in **Table 2** and **Figure 2**. AST levels after 6 months of spiomet treatment correlated negatively with the FAI (R = −0.537; P = 0.02) and FGF21 (R = −0.531; P = 0.042) levels, and positively with HOMA-IR (R = 0.546; P = 0.035). No significant association was observed in the OC subgroup. ALT levels in spiomet-treated girls also associated negatively with the FAI (R = −0.458; P = 0.014) and with HOMA-IR (R = 0.524; P = 0.014); in the OC subgroup, ALT concentrations positively correlated with SHBG (R = 0.496; P = 0.011) and METRNL (R = 0.449; P = 0.036) levels. GGT levels in OC-treated girls were also found to be strongly correlated with METRNL concentrations (R = 0.552; P = 0.004).

**Table 2:** Correlation between liver enzymes [aspartate transaminase (AST), alanine aminotransferase (ALT), and gamma-glutamyl transpeptidase (GGT)] and study variables in girls with polycystic ovary syndrome (PCOS) after 6 months on an oral contraceptive (OC) or a low-dose combination of spironolactone plus pioglitazone plus metformin (spiomet).

	AST (µkat/L)				ALT (µkat/L)				GGT (µkat/L)			
	PCOS (6 months on-treatment)				PCOS (6 months on-treatment)				PCOS (6 months on-treatment)			
	OC (N = 26)		spiomet (N = 28)		OC (N = 26)		spiomet (N = 28)		OC (N = 26)		spiomet (N = 28)	
	R	P	R	P	R	P	R	P	R	P	R	P
Weight (kg)	−0.226	0.480	0.167	0.551	0.371	0.235	0.176	0.530	0.097	0.765	0.089	0.754
Testosterone (nmol/L)	0.031	0.924	−0.439	0.102	−0.023	0.943	−0.200	0.474	0.208	0.517	0.076	0.788
SHBG (nmol/L)	0.103	0.750	0.360	0.188	**0.496**	**0.011**	0.279	0.315	0.072	0.730	0.540	0.087
FAI	0.168	0.535	−**0.537**	**0.002**	−0.346	0.271	−**0.458**	**0.014**	0.185	0.364	−0.081	0.685
Glucose (mmol/L)	0.568	0.064	0.239	0.391	−0.185	0.565	0.474	0.074	0.153	0.635	−0.041	0.886
Insulin (pmol/L)	−0.409	0.186	0.496	0.060	-0.045	0.890	0.485	0.067	−0.433	0.160	0.199	0.478
HOMA-IR	0.200	0.532	**0.546**	**0.035**	0.014	0.819	**0.524**	**0.028**	−0.375	0.229	0.198	0.477
usCRP (nmol/L)	0.456	0.136	0.553	0.073	0.406	0.190	0.461	0.082	0.154	0.756	0.151	0.442
METRNL (pg/mL)	0.255	0.359	−0.210	0.435	**0.449**	**0.036**	0.149	0.423	**0.552**	**0.004**	0.157	0.424
log DBI (ng/mL)	0.270	0.396	−0.206	0.461	−0.397	0.202	−0.321	0.244	−0.101	0.755	0.069	0.824
log FGF21 (pg/mL) ^&^	0.014	0.946	−**0.531**	**0.042**	−0.256	0.422	−0.104	0.712	0.025	0.939	0.183	0.513
Lean mass (kg)	−0.211	0.510	0.151	0.591	0.019	0.924	0.178	0.364	0.210	0.512	−0.313	0.256
Fat mass (kg)	−0.063	0.845	0.098	0.727	0.064	0.753	0.244	0.209	−0.074	0.820	0.489	0.075
Abdominal fat (kg)	−0.125	0.699	0.011	0.970	0.012	0.950	0.203	0.299	−0.254	0.425	0.133	0.635
Subcutaneous fat (cm^2^)	−0.161	0.617	−0.042	0.882	0.023	0.932	0.228	0.241	0.169	0.408	0.291	0.132
Visceral fat (cm^2^)	−0.374	0.231	0.281	0.311	0.187	0.560	0.084	0.667	−0.358	0.077	0.154	0.432
Hepatic fat (%)	0.334	0.288	−0.214	0.443	0.187	0.560	0.054	0.848	−0.233	0.251	0.237	0.221

ALT, alanine transaminase; AST, aspartate transaminase; DBI, diazepam-binding inhibitor; FAI, free androgen index; FGF21, fibroblast growth factor 21; GGT, gamma-glutamyl transferase; HOMA-IR, homeostasis model assessment insulin resistance; METRNL, meteorin-like; OC, oral contraceptive; SHBG, sex hormone-binding globulin; spiomet, spironolactone, pioglitazone, metformin; usCRP, ultrasensitive C-reactive protein.

Results are shown as R coefficients and P-values, adjusted for body mass index in multiple regression analysis. Significant values are in bold.

^&^FGF21 assessment was performed in N = 50 girls with PCOS (N = 24 OC, N = 26 spiomet).

The correlations between organokines and the study variables are shown in **Table 3**. In spiomet-treated girls, FGF21 negatively correlated with visceral fat (R = −0.750; P = 0.001).

**Table 3:** Correlations between organokines [meteorin-like (METRNL), fibroblast growth factor 21 (FGF21) and diazepam-binding inhibitor (DBI)] and study variables in girls with polycystic ovary syndrome (PCOS) after 6 months on an oral contraceptive (OC) or on a low-dose combination of spironolactone plus pioglitazone plus metformin (spiomet).

	METRNL (pg/mL)				log DBI (ng/mL)				log FGF21 (pg/mL)			
	PCOS (6 months on-treatment)				PCOS (6 months on-treatment)				PCOS (6 months on-treatment)			
	OC (N = 26)		spiomet (N = 28)		OC (N = 26)		spiomet (N = 28)		OC (N = 24)		spiomet (N = 26)	
	R	P	R	P	R	P	R	P	R	P	R	P
Weight (kg)	0.217	0.436	−0.360	0.171	−0.471	0.122	−0.122	0.554	−0.313	0.321	-0.087	0.759
Testosterone (nmol/L)	−0.154	0.584	−0.249	0.352	0.477	0.117	0.083	0.788	−0.327	0.300	0.489	0.064
SHBG (nmol/L)	0.158	0.575	−0.430	0.097	−0.150	0.642	0.107	0.729	−0.363	0.248	0.079	0.778
FAI	−0.334	0.289	0.230	0.408	0.367	0.240	−0.040	0.896	0.167	0.603	0.433	0.107
Glucose (mmol/L)	−0.169	0.548	−0.034	0.900	0.229	0.475	−0.057	0.852	−0.172	0.594	-0.012	0.968
Insulin (pmol/L)	−0.450	0.092	0.247	0.357	−0.337	0.284	−0.003	0.865	−0.216	0.499	−0.307	0.895
HOMA-IR	−0.460	0.085	0.245	0.360	-0.259	0.196	−0.001	0.985	−0.255	0.242	−0.039	0.891
usCRP (nmol/L)	0.366	0.252	−0.259	0.333	−0.045	0.825	−0.249	0.201	−0.115	0.723	−0.177	0.528
Lean mass (kg)	0.219	0.434	−0.280	0.293	−0.245	0.443	−0.049	0.873	−0.321	0.309	−0.385	0.157
Fat mass (kg)	0.034	0.905	−0.134	0.621	−0.427	0.167	−0.445	0.127	−0.029	0.930	0.377	0.166
Abdominal fat (kg)	−0.003	0.993	−0.168	0.533	−0.310	0.326	−0.464	0.111	−0.059	0.855	0.348	0.204
Subcutaneous fat (cm^2^)	−0.470	0.077	−0.066	0.983	−0.014	0.964	−0.187	0.079	−0.208	0.931	0.358	0.190
Visceral fat (cm^2^)	−0.241	0.384	0.059	0.829	−0.085	0.792	−0.341	0.255	0.274	0.389	−**0.750**	**0.001**
Hepatic fat (%)	−0.060	0.831	−0.119	0.661	−0.273	0.230	−0.133	0.664	0.096	0.767	0.208	0.458

DBI, diazepam-binding inhibitor; FAI, free androgen index; FGF21, fibroblast growth factor 21; HOMA-IR, homeostasis model assessment insulin resistance; METRNL, meteorin-like; OC, oral contraceptive; SHBG, sex hormone-binding globulin; spiomet, spironolactone, pioglitazone, metformin; usCRP, ultrasensitive C-reactive protein.

Results are shown as R coefficients and P-values, adjusted for body mass index in multiple regression analysis. Significant values are in bold.

## 4 Discussion

The present longitudinal on- and post-treatment observations in adolescent girls with PCOS and without obesity receiving either OCs or spiomet revealed that circulating levels of ALT and GGT increased only under OC intervention, indicating a stressful effect on the liver (36, 37), which nevertheless reverted upon treatment discontinuation.

Our data are in line with those of previous studies reporting the influence of OCs on liver enzymes (38) and the effects of metformin on circulating ALT and GGT (but not AST) levels (39). Indeed, serum ALT activity is considered a highly sensitive biomarker of hepatic damage, ahead of circulating AST levels, and serum GGT activity is considered an additional biomarker of liver function used to extend the information provided by ALT activity (36).

Both treatments upregulated FGF21 levels, an effect previously observed after ethinylestradiol–cyproterone acetate-based OC treatment in PCOS adolescents (35). The inverse correlation between circulating FGF21 levels and visceral fat only in spiomet-treated girls could reflect an increase in FGF21 signaling in visceral adipose tissue, followed by a more insulin-sensitive status (40). No significant associations were found between the on-treatment FGF21 changes and ALT and GGT concentrations.

To our knowledge, this is the first study in PCOS adolescents exploring the effects of two randomized treatments on the circulating concentrations of DBI, a multifunctional protein that mediates broad hepatoprotective effects (41), and METRNL, a regulatory protein involved in adipose tissue plasticity, inflammation, and cardiac function (42), recently identified as a potential hepatokine (30). DBI levels were lower in OC-treated patients than in spiomet-treated patients, but showed no correlation with indicators of hepatic damage. In contrast, METRNL levels did not differ between the two randomized subgroups but showed a strong positive association with ALT and GGT only in OC-treated PCOS girls, in whom hepatic enzymes experienced an on-treatment upward change. This finding is somewhat unexpected, as METRNL, previously considered mostly an adipokine and myokine, has been proposed to play a protective role against insulin resistance and inflammation in experimental models of obesity (43), and is usually downregulated in adult patients with obesity (44). However, our findings are consistent with a recent report pointing to METRNL as a hepatokine specifically induced by hepatic injury in obese patients (30). Considering the aforementioned positive role of METRNL in systemic metabolism, our results suggest enhanced METRNL synthesis in OC-treated girls as a reaction to the hepatic changes elicited by OC treatment. We cannot unequivocally establish that the association between METRNL levels and markers of hepatic damage corresponds to altered METRNL synthesis, specifically in the liver. However, hepatic stress may lead to increased production of protective agents, and FGF21 is an example of such a response (31). METRNL has anti-inflammatory properties and acts via the c-Kit receptor (45), which is expressed in the liver and peripheral tissues (46). Enhanced METRNL signaling may be speculated to be a compensatory mechanism intended to prevent systemic alterations, including inflammation, in response to hepatic insults. In any case, our findings suggest a potential role of METRNL as a molecular factor related to changes in liver enzymes with systemic metabolism, which warrants further investigation.

This study has several limitations. First, the small sample size and limited availability of samples precluded a longitudinal analysis of organokine concentrations that had to be restricted to a single time point of treatment. Second, access to liver biopsy samples, unfeasible for obvious ethical reasons in this type of study, would have provided particularly relevant information on the hepatic expression of METRNL in relation to OC. The strengths of the present report include the randomized study design, rather homogeneous study population, and assessment of novel organokines under two randomized treatments.

In conclusion, the pattern of circulating DBI, but not of FGF21 and METRNL, differs in adolescent girls with PCOS receiving OCs or spiomet in randomized studies. The on-treatment increase in ALT and GGT levels, occurring only in OC-treated girls, directly associated with the circulating levels of METRNL, suggesting enhanced METRNL synthesis as a reaction to the hepatic changes elicited by OC treatment.

## Data availability statement

The raw data supporting the conclusions of this article will be made available by the authors, without undue reservation.

## Ethics statement

The studies involving humans were approved by Institutional Review Board of University of Barcelona, Sant Joan de Déu University Hospital. The studies were conducted in accordance with the local legislation and institutional requirements. Written informed consent for participation in this study was provided by the participants’ legal guardians/next of kin. Written informed consent was obtained from the individual(s), and minor(s)’ legal guardian/next of kin, for the publication of any potentially identifiable images or data included in this article.

## Author contributions

CG-B: Writing – original draft, Data curation, Formal analysis, Investigation, Methodology. MP: Data curation, Formal analysis, Writing – review & editing. AN-G: Writing – review & editing, Data curation. AL-B: Conceptualization, Writing – review & editing. FZ: Conceptualization, Writing – review & editing. FV: Writing – original draft, Conceptualization, Supervision, Writing – review & editing. LI: Writing – review & editing, Conceptualization, Funding acquisition, Supervision, Writing – original draft.
