# Supplementary material for: Organokines and liver enzymes in adolescent girls with polycystic ovary syndrome during randomized treatments
Source: Front Endocrinol (Lausanne). 2024 May 16;15:1325230. doi: 10.3389/fendo.2024.1325230 (PMC11137167; doi:10.3389/fendo.2024.1325230)
Supplement: Supplementary file 1 [file DataSheet_1.pdf]

Supplementary Material

Organokines and liver enzymes in adolescent girls with polycystic ovary syndrome during randomized treatments

Cristina Garcia-Beltran, Marion Peyrou, Artur Navarro-Gascon, Abel López-Bermejo, Francis de Zegher, Francesc Villarroya*, Lourdes Ibáñez*

*** Correspondence:**
Francesc Villarroya, PhD
fvillarroya@ub.edu

Lourdes Ibáñez, MD, PhD
lourdes.ibanez@sjd.es

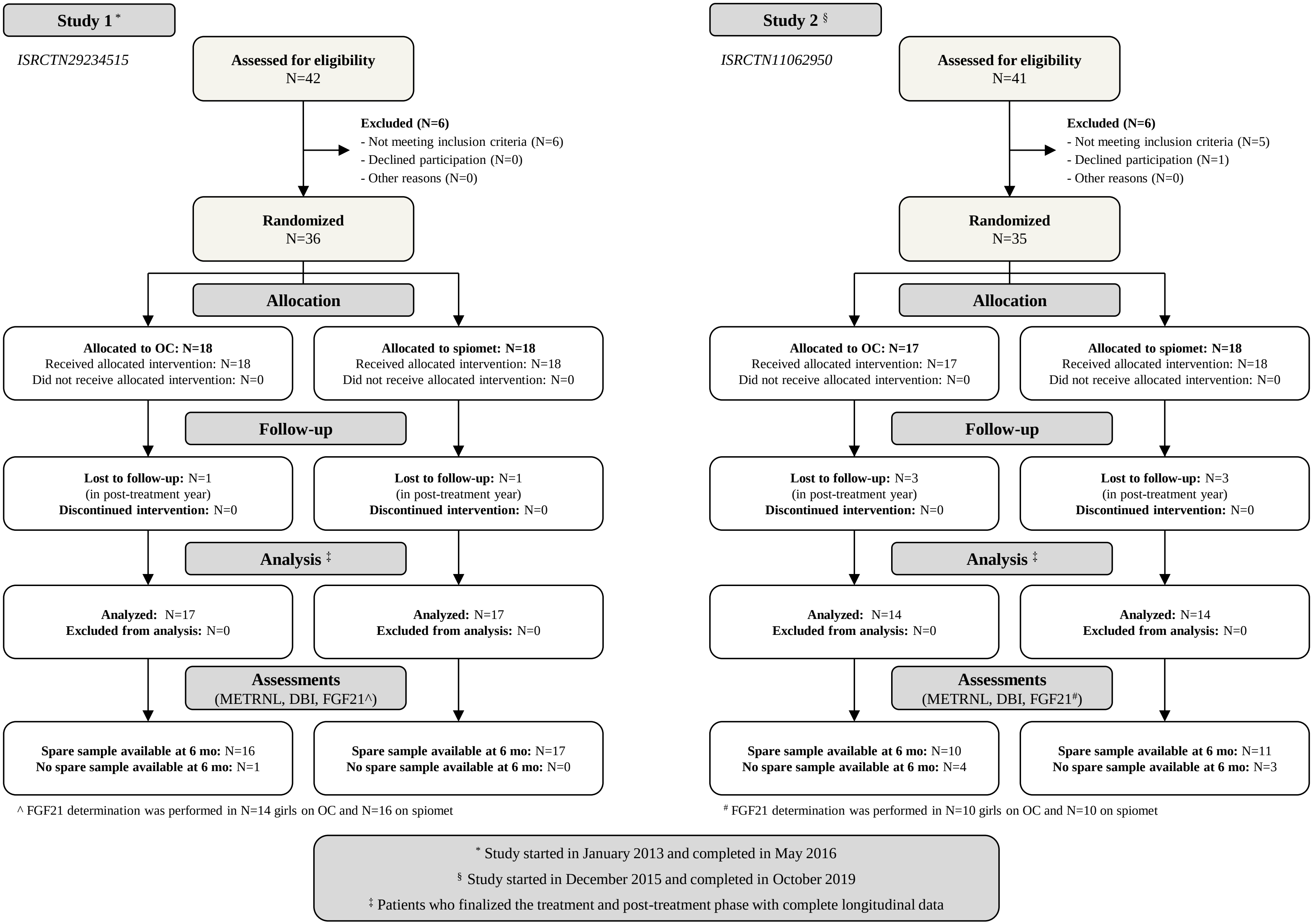

DBI, diazepam-binding inhibitor; FGF21, fibroblast growth factor 21; METRNL, meteorin-like; OC, oral contraceptive; spiomet, spironolactone plus pioglitazone plus metformin.

Supplementary Figure 1. Recruitment of the study population
